# Socioeconomic Determinants of Willingness to Pay for Emergency Public Dental Services in Saudi Arabia: A Contingent Valuation Approach

**DOI:** 10.3390/ijerph192215205

**Published:** 2022-11-17

**Authors:** Halah Saleh Hawsawi, Mustapha Immurana, Mohammed Khaled Al-Hanawi

**Affiliations:** 1 Quality and Patient Safety Department, Al-Noor Specialist Hospital, Mecca 24241, Saudi Arabia; 2 Department of Health Services and Hospital Administration, Faculty of Economics and Administration, King Abdulaziz University, Jeddah 21589, Saudi Arabia; 3 Institute of Health Research, University of Health and Allied Sciences, Private Mail Bag 31, Ho, Ghana; 4 Health Economics Research Group, King Abdulaziz University, Jeddah 21589, Saudi Arabia

**Keywords:** contingent valuation, public dental services, Saudi Arabia, socioeconomic factors, willingness to pay

## Abstract

Dental diseases remain major health problems worldwide, leading to pain, discomfort, and even death. In Saudi Arabia, public dental care services (i.e., services provided by government-owned health facilities) are provided free of charge for all Saudi citizens. However, public dental care facilities are overburdened and overcrowded, resulting in long waiting times to access dental care services. The consequent limited access to dental services can prolong discomfort and delay pain management, thereby exacerbating the suffering of patients. Therefore, the aim of this study was to examine the socioeconomic determinants of the willingness to pay for immediate public dental care in the face of a dental emergency in Saudi Arabia. A cross-sectional design was employed to obtain data from adult citizens of Saudi Arabia who were residents of the Holy City of Makkah. A pre-tested online questionnaire was used to obtain the responses from 549 individuals, selected through a snowball sampling technique, from 15 July to 10 August 2021. Descriptive statistics (frequencies and percentages), Pearson’s chi-square test, and binary probit regression were used as estimation techniques. The findings showed that 79.4% of the respondents were willing to pay for immediate public dental services, with the majority (86%) expressing a willingness to pay less than 500 Saudi Riyal (SAR). The binary probit regression estimates showed that respondents who were unemployed, those with a high school level of education or below, and those without private health insurance were less likely to be willing to pay for immediate public dental services. Thus, policies and initiatives aimed at enhancing the willingness to pay for immediate public dental services should target the unemployed, those with a high school level of education or below, and people without private health insurance.

## 1. Introduction

Although oral or dental diseases can be largely prevented, they remain major health problems for several countries, causing disfigurement, discomfort, pain, and death. Approximately 3.5 billion people are affected by oral diseases worldwide; however, treating conditions of oral health is expensive and normally not included in universal health coverage [1]. Estimates show that, globally, the direct treatment cost of dental diseases each year is USD 298 billion (corresponding to 4.6% of the global health expenditure, on average) and the indirect cost per year is USD 144 billion [2]. In the Kingdom of Saudi Arabia (KSA), the prevalence of dental diseases remains very high among children and adults. For instance, among children, the prevalence of dental caries and its associated severity is roughly 80% for primary dentition [3]. Among adults aged 30–45 years, a dental caries prevalence of up to 98% has been reported [4]. 

Although basic dental care services are provided for free by the public (government) healthcare sector in the KSA [5], these services are characterized by overload, overburden, overcrowding, shortages of providers, long waiting times, and limited treatment choices [6,7]. These factors can lead to limited access, which can delay discomfort relief and effective pain management, especially during emergencies, hence exacerbating the suffering of patients. There is therefore a need for a new treatment approach as an alternative to the services provided by the public healthcare sector that would make it possible for people to access dental care at any time, based on need. However, such a treatment approach would come at a cost; hence, its success is dependent on the willingness of people to pay for such services, especially in emergency situations. Thus, willingness to pay (WTP) is one of the best means of assessing the acceptability of novel treatment approaches [8].

To this end, several studies have been conducted to evaluate the WTP for dental services [8,9,10,11,12,13,14,15,16,17,18,19]. Among these studies, to the best of our knowledge, only a few were devoted to the KSA [8,10,19]. The study by Al Garni et al. [8] sampled 100 respondents who had at least one missing tooth and found that 67% of them were willing to pay the median price (SAR 3000) for an implant. In addition, the study by Fatani and Al-Yousef [10] examined the WTP for orthodontic treatment of children by sampling 171 parents. While they found that 71.6% of the parents disagreed with the fairness of the median WTP amount of SAR 10,000, among those who agreed that the median amount was fair, 71.4% indicated their WTP for more advanced dental treatment for their children [10]. Moreover, the study of 200 patients in the KSA by Linjawi et al. [19] found that 47.5% of the respondents indicated their ability to pay for additional procedures to lessen the treatment time for orthodontic procedures. However, none of the studies on the KSA considered WTP for dental care due to unexpected dental pain occurring outside of normal service hours or days. Examining WTP for urgent dental pain is important because the 74th session of the World Health Assembly held in 2021 approved an oral health resolution that, among other proposals, recommends timely and inclusive oral care [1]. 

Based on this background and gaps in the literature, this study examined the socioeconomic determinants of WTP for immediate public dental services in the case of a sudden dental pain occurring outside normal services hours or days among 549 adults in Makkah City of the KSA, using a contingent valuation approach. This approach facilitates using surveys to examine the economic value of goods and services not ordinarily found on the market, and thus aids in revealing the willingness of individuals to pay for such goods and services, as well as the amount they are willing to pay [20,21]. Therefore, the findings of this study can offer important guidance in informing the acceptability and success of new treatment models aimed at providing immediate dental services for patients outside regular services hours or days, ultimately reducing the delays associated with managing urgent dental pain and discomfort.

## 2. Materials and Methods

### 2.1. Study Design, Setting, and Sample

A cross-sectional design was employed from 15 July to 10 August 2021 to obtain data from adult citizens of Saudi Arabia who were residents of Makkah City in the Makkah region, one of the two holy sites in the KSA. Makkah City was selected because of its cosmopolitan nature, encompassing a sample of people with diverse backgrounds. This study included participants who were Saudi citizens, residents of Makkah city, and aged 18 years and above. The study was limited to only Saudi citizens because non-Saudi nationals are not eligible to access free public health services [22]. According to the latest KSA census in 2019, Makkah had a total population of 855,805 Saudi citizens [23]. The minimum sample size necessary for the study was calculated to be 384 (margin of error of 5%, confidence level of 95%, and 50% response distribution, based on the total 855,805 Saudi citizens in the region). However, to curtail the rate of nonresponses and unusable data, over 600 respondents were targeted.

The questionnaire was developed based on previous studies [9,22]. The questionnaire was initially drafted in English by H.S.H. and was translated from English to Arabic by M.K.A. The questionnaire was then back translated to English by another colleague to ensure the intended meaning of the content. Moreover, the questionnaire was pre-tested, and all errors were rectified before final data collection. Given the restrictions instituted in the country during the study period to curtail the spread of COVID-19 in the midst of the pandemic, the data were collected (in the Arabic language) via an online self-reported questionnaire using Google Forms. 

A snowball sampling technique was used in selecting the potential respondents. This sampling technique was chosen because of the restrictions on movement imposed by the government during the COVID-19 pandemic, which would have made it very difficult to use a probability sampling technique. To this end, we obtained phone contacts of dental patients from major health facilities in Makkah City. We then contacted these individuals via WhatsApp to participate in the study by sending them the Google Forms link. The patients were also kindly requested to forward the Google Forms link to their WhatsApp contacts. A total of 652 participants completed the questionnaire. However, due to incomplete responses and missing data, the study restricted the analysis to the sample of 549 respondents who provided complete information.

### 2.2. Variables and Data Analysis Techniques

The dependent variables used in this study were WTP and the highest amount respondents were willing to pay for immediate public dental service. These variables were obtained using a contingent valuation approach by posing the scenario below to the respondents:

Imagine that you or one of your family members suddenly complain of tooth pain and you are seeking to see the doctor in the public dental services, but it is a weekend or a holiday and you have to wait for two days or more; the pain gets worse, and you need to visit the dentist urgently. So, you go to a public dental facility and find that the service will be provided to you at a fee. Would you be willing to pay to see the dentist immediately, or, at the latest, the day after you first noticed the pain? If yes, what would be the highest amount you would be willing to pay?

With regard to the contingent valuation approach, we minimized hypothetical bias using the familiarity approach by first contacting patients who were familiar with dental care. Thus, according to Mitchell and Carson [21], the more familiar individuals are with a good or service, the lower the likelihood of hypothetical bias in a contingent valuation response. Moreover, the collection of data via a non-face-to-face (online) approach and the anonymity of responses provided by the respondents helped in reducing the guilt effect since responses could not be linked to the respondents. Additionally, the scenario narrated to the respondents has the potential to reduce any indignation effect because it does not suggest scrapping the free public dental services, but rather, inquiries about the respondents’ WTP for immediate public dental service during a weekend or holiday.

The response to the WTP question was binary (yes or no). Similarly, the response to the highest amount respondents would be willing to pay was categorized as less than SAR 500 or SAR 500 and above. The independent variables used in this study were age, gender, marital status, educational level, employment status, monthly income level, and private health insurance status. All the independent variables were categorical and hence, were treated as dummy variables. The respondents were not restricted to household heads; therefore, the WTP, the maximum amount the respondent was willing to pay, and monthly income of the respondent were all requested at the individual level.

With regard to the estimation techniques, simple frequencies and percentages were used to analyze the WTP, as well as the maximum amount the respondent was willing to pay, and the Pearson’s chi-square (χ^2^) test was used to determine the extent of the associations between the socioeconomic (independent) variables and WTP, as well as the maximum amount the respondents were willing to pay. The binary probit regression model was used to examine the socioeconomic determinants of WTP and the highest amount respondents were willing to pay for immediate public dental services. The binary probit regression model was selected for this analysis given the binary nature of the dependent variables. The ordinary least-squares regression was not used because it is unable to capture the discrete nature of the dependent variables [24]. To facilitate a more intuitive interpretation of the regression results, we presented the average marginal effects (AMEs) of the probit regression [25], in addition to the estimated coefficients. All analyses were conducted using STATA software version 14.0 (StataCorp LP, College Station, TX, USA).

## 3. Results

### 3.1. Socio-Economic Characteristics of the Study Participants

With regard to the background of the sampled respondents, 32.6% were aged 30–39 years, followed by those aged 18–29 years (24.2%). The majority of the sampled respondents were women (61.6%), married (63.8%), and college/university degree holders (63.0%) (Table 1).

Government and non-government employees formed the majority of the respondents (63.8%). Only 6.6% of the respondents had income levels of SAR 20,000 or more, and the majority of the respondents did not have private health insurance (69.6%). Concerning the dependent variables, the majority of the respondents (79.4%) were willing to pay for immediate public dental services, with 86% of them willing to pay less than SAR 500 (Table 1).

### 3.2. Bivariate Analysis of Socioeconomic Factors, WTP, and the Amount Respondents Were Willing to Pay for Immediate Public Dental Services in an Emergency

Among the age groups, those 30–39 years old had the highest percentage of respondents who indicated that they were willing to pay for immediate public dental services (32.1%). The majority of those who were willing to pay for immediate public dental services were women (59.6%); however, 69.0% of those who were not willing to pay were also women. Similarly, the majority of those who were willing to pay for immediate public dental services were married (65.1%) and had a college/university degree (62.6%) (Table 2).

There was a statistically significant association between educational level and WTP for immediate public dental services (χ^2^ = 7.67, *p* = 0.02). Moreover, concerning the association between employment status and WTP for immediate public dental services, government employees included the highest percentage of people who were willing to pay (44.5%), followed by non-government employees (22.0%). Hence, we found a statistically significant difference among the various employment categories with regard to WTP for immediate public dental services (χ^2^ = 21.17, *p* = 0.001).

The majority of those who were willing to pay for immediate public dental service were those not having private health insurance (66.7%), showing a statistically significant association (χ^2^ = 8.06, *p* = 0.005) (Table 2). As shown in Table 3, among the socioeconomic variables, only marital status, educational level, and employment status showed significant differences between categories with respect to the maximum amount respondents were willing to pay.

### 3.3. Regression Results

Table 4 and Table 5 present the results of the binary probit regression estimates of the socioeconomic determinants of WTP for immediate public dental services and the maximum amount respondents were willing to pay, respectively. As shown in Table 4, the respondents with a college/university degree or a postgraduate degree were more willing to pay for immediate public dental services relative to those with a high school level of education or below. Specifically, respondents with a college/university degree and those with a postgraduate degree were 11% (AME: 0.11, *p* < 0.1) and 19% (AME: 0.19, *p* < 0.01) more willing to pay for immediate public dental services, respectively, relative to their counterparts with a high school level of education or below.

With regard to employment status, those who were students, government and non-government employees, and retirees were found to be more willing to pay for immediate public dental services relative to their unemployed counterparts. Specifically, respondents who were students, government employees, non-government employees, and retirees were 21% (AME: 0.21, *p* < 0.01), 17% (AME: 0.17, *p* < 0.05), 18% (AME: 0.18, *p* < 0.05), and 20% (AME: 0.20, *p* < 0.05) more willing to pay for immediate public dental services, respectively, relative to their unemployed counterparts. Private health insurance was also found to have a positive association with WTP for immediate public dental services. Specifically, respondents with private health insurance were 9% (AME: 0.09, *p* < 0.05) more willing to pay for immediate public dental services relative to those without private health insurance (Table 4).

Turning to the socioeconomic determinants of the maximum amount respondents were willing to pay for immediate public dental service (Table 5), we found that respondents aged 50 years and above were 12% (AME: −0.12, *p* < 0.1) less willing to pay SAR 500 and above, relative to those who were 18–29 years old. Moreover, married respondents were found to be 9% (AME: −0.09, *p* < 0.05) less willing to pay SAR 500 and above for immediate public dental service relative to their unmarried counterparts. Similarly, respondents with a college/university degree and those with a postgraduate degree were 15% (AME: −0.15, *p* < 0.05) and 13% (AME: −0.13, *p* < 0.1) less willing to pay SAR 500 and above for immediate public dental services, respectively, relative to their counterparts with a high school level of education or below (Table 5).

## 4. Discussion

This study examined the socioeconomic determinants of WTP for immediate public dental services in the face of dental emergencies, as well as the maximum amount individuals were willing to pay among residents of Makkah City in the KSA. Identifying the factors that determine WTP for immediate public dental services is important to guide the introduction and acceptability of new treatment approaches aimed at dealing with urgent dental cases outside the regular working hours and days of public dental services. We found that the majority of the respondents were willing to pay for public dental services in the case of an emergency (79.4%), which is very encouraging. This finding is similar to those of prior studies [8,10,11,17]. For instance, Al Garni et al. [8] found that 67% of respondents were willing to pay for implants in Riyadh. Similarly, Fatani and Al-Yousef [10] found that 71.4% of parents in Saudi Arabia were willing to pay for more advanced dental treatment for their children.

Educational level was found to play a role in WTP for immediate public dental services in both the bivariate and regression analyses. Since those with a college/university degree and postgraduate degrees are more likely to appreciate the importance of healthcare, as well as to be gainfully employed relative to those with a high school level of education or below [26], it is not implausible that they would be more willing to pay for public dental services. In fact, the willingness of those with a postgraduate education to pay for public dental services (19%) was even greater than that of respondents with a college/university degree (11%), which highlights the role of a rising level of education with regard to WTP for immediate public dental services. Therefore, initiatives aimed at enhancing WTP for dental services should target those with a high school level of education or below. Our findings are in line with those of Srivastava et al. [13], who found higher education (university graduate or higher) to be positively associated with WTP for dental care among individuals in Canada. Al-Hanawi et al. [27] also found that individuals with more years of education were more likely to have higher WTP for most indicators regarding improved public health services in Saudi Arabia.

Notwithstanding, among those who were willing to pay for immediate public dental services, respondents with college/university and postgraduate degrees were less willing to pay SAR 500 and above for immediate public dental services relative to their counterparts with a high school level of education or below. This could be due to the fact that respondents with higher level of education are more likely to have subscribed to health insurance schemes, and hence, they expect that medical expenses will be covered by such plans. In fact, our data confirm this speculation, since 127 of the 167 respondents with private health insurance (76.05%) had college/university or postgraduate degrees. This is further supported by the negative relationship, albeit statistically insignificant, between the willingness to pay SAR 500 and above and holding private health insurance.

Given that people without employment are more likely to have lower incomes, it was not surprising that they were less likely to be willing to pay for immediate public dental services relative to those who were employed (government and non-government employees). In fact, higher income has been found to be positively associated with high ability to pay for unexpected medical expenses among adults in Finland [9]. A positive association between higher income and WTP for dental care was also reported in Canada [13]. Moreover, those employed would have a higher opportunity cost if they are unable to go to work as a result of ill health, leading to a loss in earnings [26]. This would increase the WTP for immediate public dental services among those employed relative to those who are unemployed.

Our findings point to the likelihood of widening inequities between those with high socioeconomic status (the employed and those with a higher level of education) and those with low socioeconomic status (the unemployed and the less educated) regarding the WTP for immediate public dental services. Therefore, there is a need to institute measures that would ensure that only those who are capable of paying for such immediate dental services are required to pay, while the less advantaged patients are offered such services at subsidized rates or for free.

Last but not the least, since those with private health insurance may perceive healthcare to be more valuable as compared with those without private health insurance, it is not surprising that the former group was more willing to pay for immediate public dental services. In addition, those with private health insurance may also be more likely to have the financial resources to pay for immediate public dental services than those without private health insurance, given that private health insurance policies are usually expensive. Our findings highlight the importance of socioeconomic factors in determining WTP for immediate public dental services during an emergency. Specifically, our results indicate that policies aimed at enhancing the WTP for immediate public dental services should focus on those with a high school level of education or below, the unemployed, and those without private health insurance.

However, our study is not without limitations. Since we used only respondents from Makkah City and WhatsApp alone as a means to collect data, caution must be exercised in extending the findings to represent the whole country. In particular, the use of WhatsApp excludes the perceptions of individuals who do not use this social networking platform and those who are illiterate; hence, the findings may not be a true reflection of the WTP for immediate public dental services and the maximum amount patients are willing to pay among more vulnerable groups, such as the illiterate. Similarly, the use of self-reported data is likely to be influenced by the biases of respondents, and the snowball sampling technique adopted by this study is likely to lead to a homogenous and less representative sample. In addition, given that this study was a one-time survey with a limited scenario, we were unable to determine the embedding effect, hence the consistency of the contingent valuation estimates using either an internal or external scope test. Moreover, our study does not reveal how respondents perceived their oral health, which could have been an important factor in determining the WTP for immediate public dental services. Additionally, we did not distinguish between respondents who were dental patients at the time of the study and those who were not in order to determine the effect of this condition on WTP. Nonetheless, being a current dental patient could be a potential source of bias. We therefore recommend that future studies focus on addressing some of these limitations.

## 5. Conclusions

Public dental services in the KSA are characterized by overload, overburden, overcrowding, shortages of providers, long waiting times, and limited treatment choices, which together can lead to limited access, thereby extending the period of discomfort while delaying pain management. Therefore, there is a need for alternative models that provide dental care services anytime they are needed. Nonetheless, such alternative models come at a personal cost. We therefore examined the socioeconomic determinants of WTP for immediate public dental care in the face of dental emergencies among adult citizens of Saudi Arabia residing in Makkah. Our findings show that respondents with a high school level of education or below, the unemployed, and those without private health insurance were less willing to pay for immediate public dental services in an emergency situation. These findings suggest that policies and initiatives aimed at enhancing WTP for immediate public dental services should target the unemployed, those with a high school level of education or below, and people without private health insurance.

## Figures and Tables

**Table 1:** Socioeconomic characteristics of respondents.

Variable	Frequency (*n* = 549)	%
**Age (years)**		
18–29	133	24.2
30–39	179	32.6
40–49	126	23.0
≥50	111	20.2
**Gender**		
Male	211	38.4
Female	338	61.6
**Marital status**		
Unmarried	199	36.2
Married	350	63.8
**Educational level**		
High school or below	97	17.7
College/university degree	346	63.0
Postgraduate degree	106	19.3
**Employment status**		
Unemployed	87	15.8
Student	44	8.0
Self-employed	12	2.2
Government employee	241	43.9
Non-government employee	109	19.9
Retired	56	10.2
Monthly income (in SAR) *		
˂5000	159	29.0
5000 to ˂10,000	127	23.1
10,000 to ˂15,000	142	25.9
15,000 to ˂20,000	85	15.5
≥20,000	36	6.6
**Private health insurance**		
No	382	69.6
Yes	167	30.4
**Willingness to pay**		
No	113	20.6
Yes	436	79.4
Amount willing to pay (in SAR) **		
˂500	375	86.0
≥500	61	14.0

* 1 SAR = USD 0.27; ** This included only respondents who indicated they were willing to pay (*n* = 436).

**Table 2:** Bivariate analysis of socioeconomic factors and willingness to pay for immediate public dental services.

Variable	Willingness to Pay			χ^2^	*p*-Value
	No	Yes	Total		
	*n* (%)	*n* (%)	*n* (%)		
**Age (years)**				0.930	0.818
18–29	24 (21.2)	109 (25.0)	133 (24.2)		
30–39	39 (34.5)	140 (32.1)	179 (32.6)		
40–49	28 (24.8)	98 (22.5)	126 (23.0)		
≥50	22 (19.5)	89 (20.4)	111 (20.2)		
**Gender**				3.347	0.067
Male	35 (31.0)	176 (40.4)	211 (38.4)		
Female	78 (69.0)	260 (59.6)	338 (61.6)		
**Marital status**				1.759	0.185
Unmarried	47 (41.6)	152 (34.9)	199 (36.2)		
Married	66 (58.4)	284 (65.1)	350 (63.8)		
**Educational level**				7.665	0.022
High school or below	27 (23.9)	70 (16.1)	97 (17.7)		
College/university degree	73 (64.6)	273 (62.6)	346 (63.0)		
Postgraduate degree	13 (11.5)	93 (21.3)	106 (19.3)		
**Employment status**				21.170	0.001
Unemployed	32 (28.3)	55 (12.6)	87 (15.8)		
Student	7 (6.2)	37 (8.5)	44 (8.0)		
Self-employed	4 (3.5)	8 (1.8)	12 (2.2)		
Government employee	47 (41.6)	194 (44.5)	241 (43.9)		
Non-government employee	13 (11.5)	96 (22.0)	109 (19.9)		
Retired	10 (8.8)	46 (10.6)	56 (10.2)		
**Monthly income (SAR)**				5.731	0.220
˂5000	42 (37.2)	117 (26.8)	159 (29.0)		
5000 to ˂10,000	21 (18.6)	106 (24.3)	127 (23.1)		
10,000 to ˂15,000	29 (25.7)	113 (25.9)	142 (25.9)		
15,000 to ˂20,000	16 (14.2)	69 (15.8)	85 (15.5)		
≥20,000	5 (4.4)	31 (7.1)	36 (6.6)		
**Private health insurance**				8.060	0.005
No	91 (80.5)	291 (66.7)	382 (69.6)		
Yes	22 (19.5)	145 (33.3)	167 (30.4)		
**Total**	113 (20.6)	436 (69.6)	549 (100)		

**Table 3:** Bivariate analysis of socioeconomic factors and the maximum amount respondents were willing to pay for immediate public dental services.

Variable	Amount Willing to Pay (in SAR)			χ^2^	*p*-Value
	˂500 SAR	≥500 SAR	Total		
	*n* (%)	*n* (%)	*n* (%)		
**Age (years)**				1.697	0.638
18–29	90 (24.0)	19 (31.1)	109 (25.0)		
30–39	121 (32.3)	19 (31.1)	140 (32.1)		
40–49	85 (22.7)	13 (21.3)	98 (22.5)		
≥ 50	79 (21.0)	10 (16.4)	89 (20.4)		
**Gender**				1.040	0.308
Male	155 (41.3)	21 (34.4)	176 (40.4)		
Female	220 (58.7)	40 (65.6)	260 (59.6)		
**Marital status**				5.020	0.025
Unmarried	123 (32.8)	29 (47.5)	152 (34.9)		
Married	252 (67.2)	32 (52.5)	284 (65.1)		
**Educational level**				5.486	0.064
High school or below	54 (14.4)	16 (26.2)	70 (16.1)		
College/university degree	240 (64.0)	33 (54.1)	273 (62.6)		
Postgraduate degree	81 (21.6)	12 (19.7)	93 (21.3)		
**Employment status**				9.812	0.081
Unemployed	50 (13.3)	5 (8.2)	55 (12.6)		
Student	28 (7.5)	9 (14.8)	37 (8.5)		
Self-employed	6 (1.6)	2 (3.3)	8 (1.8)		
Government employee	162 (43.2)	32 (52.5)	194 (44.5)		
Non-government employee	89 (23.7)	7 (11.5)	96 (22.0)		
Retired	40 (10.7)	6 (9.8)	46 (10.6)		
**Monthly income (SAR)**				1.248	0.870
˂5000	102 (27.2)	15 (24.6)	117 (26.8)		
5000 to ˂10,000	89 (23.7)	17 (27.9)	106 (24.3)		
10,000 to ˂15,000	98 (26.1)	15 (24.6)	113 (25.9)		
15,000 to ˂20,000	58 (15.5)	11 (18.0)	69 (15.8)		
≥20,000	28 (7.5)	3 (4.9)	31 (7.1)		
**Private health insurance**				2.400	0.121
No	245 (65.3)	46 (75.4)	291 (66.7)		
Yes	130 (34.7)	15 (24.6)	145 (33.3)		
**Total**	375 (86.0)	61 (14.0)	436 (100)		

**Table 4:** Socioeconomic determinants of willingness to pay for immediate public dental services.

Variable	Model 1	Model 2
	Coefficients (SE)	Average Marginal Effects (SE)
**Age (years)**		
18–29	Ref	Ref
30–39	−0.20 (0.20)	−0.05 (0.05)
40–49	−0.25 (0.23)	−0.07 (0.06)
≥50	−0.16 (0.28)	−0.04 (0.07)
**Gender**		
Male	Ref	Ref
Female	−0.04 (0.16)	−0.01(0.04)
**Marital status**		
Unmarried	Ref	Ref
Married	0.20 (0.15)	0.05 (0.04)
**Educational level**		
High school or below	Ref	Ref
College/university degree	0.37 ** (0.18)	0.11 * (0.06)
Postgraduate degree	0.71 *** (0.24)	0.19 *** (0.06)
**Employment status**		
Unemployed	Ref	Ref
Student	0.74 ** (0.30)	0.21 *** (0.08)
Self-employed	0.05 (0.43)	0.02 (0.15)
Government employee	0.56 ** (0.26)	0.17 ** (0.08)
Non-government employee	0.58 ** (0.27)	0.18 ** (0.08)
Retired	0.65 * (0.34)	0.20 ** (0.10)
**Monthly income (SAR)**		
˂5000	Ref	Ref
5000 to ˂10,000	0.07 (0.22)	0.02 (0.06)
10,000 to ˂15,000	−0.12 (0.25)	−0.03 (0.07)
15,000 to ˂20,000	−0.14 (0.27)	−0.04 (0.07)
≥20,000	0.06 (0.34)	0.01 (0.08)
**Private health insurance**		
No	Ref	Ref
Yes	0.36 ** (0.18)	0.09 ** (0.04)
**Constant**	−0.00 (0.27)	
**Observations**	549	549

Note: Average marginal effects (AMEs) were used; SE: standard errors; * *p* < 0.1, ** *p* < 0.05, *** *p* < 0.01.

**Table 5:** Socioeconomic determinants of the maximum amount respondents were willing to pay for immediate public dental services.

Variable	Model 1	Model 2
	Coefficients (SE)	Average Marginal Effects (SE)
**Age (years)**		
18–29	Ref	Ref
30–39	−0.15 (0.26)	−0.04 (0.06)
40–49	−0.25 (0.31)	−0.06 (0.07)
≥50	−0.60 * (0.36)	−0.12 * (0.07)
**Gender**		
Male	Ref	Ref
Female	0.24 (0.19)	0.05 (0.04)
**Marital status**		
Unmarried	Ref	Ref
Married	−0.39 ** (0.19)	−0.09 ** (0.04)
**Educational level**		
High school or below	Ref	Ref
College/university degree	−0.60 *** (0.23)	−0.15 ** (0.06)
Postgraduate degree	−0.53 * (0.28)	−0.13 * (0.07)
**Employment status**		
Unemployed	Ref	Ref
Student	0.26 (0.38)	0.05 (0.07)
Self-employed	0.98 * (0.58)	0.24 (0.17)
Government employee	0.49 (0.38)	0.10 (0.07)
Non-government employee	−0.12 (0.39)	−0.02 (0.05)
Retired	0.51 (0.47)	0.10 (0.10)
**Monthly income (SAR)**		
˂5000	Ref	Ref
5000 to ˂10,000	0.35 (0.31)	0.07 (0.06)
10,000 to ˂15,000	0.19 (0.34)	0.03 (0.06)
15,000 to ˂20,000	0.53 (0.35)	0.11 (0.07)
≥20,000	0.04 (0.43)	0.01 (0.07)
**Private health insurance**		
No	Ref	Ref
Yes	−0.03 (0.22)	−0.01 (0.05)
**Constant**	−0.00 (0.27)	
**Observations**	436	436

Note: Average marginal effects (AMEs) were used; SE: standard errors; * *p* < 0.1, ** *p* < 0.05, *** *p* < 0.01.

## Data Availability

The datasets generated and/or analyzed during the current study are not publicly available due to privacy and confidentiality agreements, as well as other restrictions, but are available from the corresponding author on reasonable request.
